# Carbon-Based Transducers for Solid-Contact Calcium Ion-Selective Electrodes: Mesopore and Nitrogen-Doping Effects

**DOI:** 10.3390/membranes12090903

**Published:** 2022-09-19

**Authors:** Yirong Zhang, Yitian Tang, Rongfeng Liang, Lijie Zhong, Jiexian Xu, Huici Lu, Xiaofeng Xu, Tingting Han, Yu Bao, Yingming Ma, Shiyu Gan, Li Niu

**Affiliations:** Guangzhou Key Laboratory of Sensing Materials & Devices, Center for Advanced Analytical Science, School of Chemistry and Chemical Engineering, Guangzhou University, Guangzhou 510006, China

**Keywords:** ion-selective electrode, potentiometric sensors, mesopore structure, N-doping, water layer

## Abstract

Solid-contact ion-selective electrodes (SC-ISEs) exhibit great potential in the detection of routine and portable ions which rely on solid-contact (SC) materials for the transduction of ions to electron signals. Carbon-based materials are state-of-the-art SC transducers due to their high electrical double-layer (EDL) capacitance and hydrophobicity. However, researchers have long searched for ways to enhance the interfacial capacitance in order to improve the potential stability. Herein, three representative carbon-based SC materials including nitrogen-doped mesoporous carbon (NMC), reduced graphene oxide (RGO), and carbon nanotubes (CNT) were compared. The results disclose that the NMC has the highest EDL capacitance owing to its mesopore structure and N-doping while maintaining high hydrophobicity so that no obvious water-layer effect was observed. The Ca^2+^-SC-ISEs based on the SC of NMC exhibited high potential stability compared with RGO and CNT. This work offers a guideline for the development of carbon-material-based SC-ISEs through mesoporous and N-doping engineering to improve the interfacial capacitance. The developed NMC-based solid-contact Ca^2+^-SC-ISE exhibited a Nernstian slope of 26.3 ± 3.1 mV dec^−1^ ranging from 10 μM to 0.1 M with a detection limit of 3.2 μM. Finally, a practical application using NMC-based SC-ISEs was demonstrated through Ca^2+^ ion analysis in mineral water and soil leaching solutions.

## 1. Introduction

Ion-selective electrodes (ISEs) act as a type of classic electrochemical sensor that enable the detection and analyses of over 60 ions [1,2,3,4]. However, traditional liquid-contact ISEs have encountered difficulties in miniaturization and instrumentation [5,6]. To overcome this challenge and satisfy practical requirements, solid-contact ion-selective electrodes (SC-ISEs) have been proposed, which extend the application to ion detection in complex environments, for example, marine salts [7,8,9,10], human biofluids [11,12,13], and even biomolecules [14,15,16].

Advances in SC-ISEs stem from the development of state-of-the-art SC materials [17], which involves the two core issues of interfacial capacitance and hydrophobicity [18]. The need for reasonable consideration of capacitance and hydrophobicity was expounded upon in our previous report [19]. In principle, a high interfacial capacitance reduces the effect of ion flux and thus achieves the purpose of stabilizing the potential. Currently, a variety of SC materials have been developed, including conductive polymers (CPs) [20,21,22,23,24], carbon-based materials [17,18], transition metal oxides [25] or sulfides [26], redox couples [27,28,29,30,31] and noble-metal nanoparticles [32,33,34,35]. Among these, CPs bear a large redox capacitance but face interference from water layer and gas effects [36]. Attempts at overcoming this issue have included adjusting the thickness of the CPs [37] and designing hydrophobic CPs [38,39]. Noble metals have a relatively high application value. Carbon-based materials are the most widely used SC materials due to their high electrical double-layer (EDL) capacitance and hydrophobicity. For example, heteroatomic doping and pore-creating engineering present effective procedures for improving the EDL capacitance of carbon materials, particularly in the field of energy storage [40]. A few typical carbon materials, for example, porous carbon [41,42,43,44,45], graphene [46,47,48,49,50], carbon nanotube [51,52,53,54,55] have been used as SC materials in SC-ISEs. However, there remains a lack of guidelines in the field of SC-ISEs with regard to improving the interfacial capacitance for carbon-based SC materials.

In this work, three representative carbon materials were chosen for the Ca^2+^-SC-ISEs including nitrogen-doped mesoporous carbon materials (NMC), reduced graphene oxide materials (RGO) and carbon nanotube materials (CNT). The results demonstrate that the NMC bear the highest capacitance due to mesoporous and N-doping effects. The SC-ISEs based on the SC of NMC showed no obvious water-layer effect and exhibited potential stability compared with RGO and CNT. Finally, the Ca^2+^ ion present in mineral water and soil-leaching solutions was detected for a proof-of-concept practical application.

## 2. Materials and Methods

### 2.1. Materials

Sodium tetrakis [3,5-bis(trifluoromethyl)phenyl] borate (NaTFPB), 2-nitrophenyl octyl ether (NPOE), high-molecular-weight poly (vinyl chloride) (PVC), tetrahydrofuran (THF), calcium ionophore IV, 4-aminoantipyrine, perfluorinated resin solution (Nafion 1100 W-5 wt%) and ethyl alcohol (anhydrous) were purchased from Sigma-Aldrich. Silica was purchased from Cabot. N-methyl-2-pyrrolidone (NMP) and iron nitrate were obtained from Macklin. All aqueous solutions were prepared with ultrapure water (>18.2 MΩ cm).

The original soil samples were collected from five different locations near Guangzhou Higher Education Mega Center. The collected 10 g soil samples were mixed with 70 mL ultrapure water and ultrasonicated for 1 h. The resulting mixture was centrifuged (10 min at 10,000 rpm) to collect the solution. Finally, the obtained solution was stored at 4 °C in a refrigerator for use. In addition, five different brands of mineral water were randomly purchased from the market for testing.

### 2.2. Synthesis of Materials

Chemically reduced graphene oxide (RGO) was synthesized via hydrothermal treatment of dialysis-purified graphene oxide (GO) at 160 °C for 3 h, followed by filtration and then freeze-dried under vacuum to obtain the samples. Graphene oxide (GO) was synthesized by the generally used oxidation of graphite. Carbon nanotubes (CNT) were obtained from XFNANO (Nanjing, China). The nitrogen-doped mesoporous carbon materials (NMC) were prepared using a silica template method. Briefly, 0.5 g of silica was first dispersed in water by ultrasonication. Next, 0.5 g of 4-aminoantipyrine was added into the silica solution for mixing. After that, 0.5 g of iron nitrate as the polymerization inducer was added and ultrasonicated for around 3 h. The mixture solution was dried in an oven at 85 °C overnight. The powder was placed under heat treatment at 800 °C for 3 h under an Ar atmosphere. Finally, the product was etched in the HF solution to remove iron particles and silica to form the NMC.

### 2.3. Material Characterizations

Brunauer–Emmett–Teller (BET) and Barrett–Joyner–Halenda (BJH) models were applied to extract the specific surface area and pore-size distribution data, respectively. Scanning electron microscopy (SEM) using a Phenom Nano SEM (Phenom Scientific, Tykyo, Japan) at 10 KV and Transmission electron microscopy (TEM) using a JEOL JEM-2100F TEM (JEOL, Tykyo, Japan) at 200 KV were carried out to analyze the morphology of three carbon materials. Contact angle tests were conducted using a contact angle apparatus (Zhijia Equipment, Shenzhen, China). X-ray diffraction (XRD) measurements were performed via Smartlab9K (Rigaku, Tykyo, Japan) with Cu-Kα as the X-ray source. X-ray photoelectron spectroscopy (XPS) data were gathered by Thermo Scientific ESCALAB 250Xi through Al Kα ray at 1486.6 eV (Waltham, MA, USA). Raman spectra were measured by a HORIBA LabRAM HR 800 with an excitation wavelength of 514 nm (HORIBA, Paris, France). The Ion Chromatography (IC) tests were performed using CIC-D120 (Qingdao Shenghan, Co., Ltd., Qingdao, China). 

### 2.4. Electrode Pretreatment

Glassy carbon electrodes (GCE) with a diameter of 5 mm were polished with 0.3 μm alumina powder and ultrasonically washed in water and ethanol (95%), respectively, and dried by N_2_ flow. The NMC, RGO and CNT solutions contain 10 mg of the sample, 250 μL of Nafion, and 750 μL of ethyl alcohol (NMP instead of RGO), which were sonicated for several hours to ensure complete dispersion. Then, the solution was drop-casted on GCE and dried at 60 °C with 1 mg cm^−2^ of mass loading.

The Ca^2+^-ISM cocktail was prepared using 3 mL THF with 117.72 mg of PVC (32.7 wt%), 235.44 mg of NPOE (65.4 wt%), 4.68 mg Calcium ionophore IV (1.3 wt%), and 2.16 mg KTPFB (0.6 wt%). Then, 50 μL of the cocktail was cast on the carbon-material based SC layer and dried at room temperature for 3 h.

### 2.5. Electrochemical Measurements

Cyclic voltammetry was tested in potential range from 0.1 to 0.6 V at a scan rate of 5 mV/s. Charge–discharge test with a current density of 0.25 A/g was performed in 0.1 M CaCl_2_ to evaluate the capacitance of NMC, RGO and CNT. Constant-current chronopotentiometry was performed for evaluation of the capacitance of corresponding SC-ISEs with an applied constant current of ±1 nA for 100 s in 0.1 M CaCl_2_. The above tests were conducted via a CHI660E electrochemical workstation (Shanghai CHI Apparatus Corporation, Shanghai, China) three-electrode system and Gamry reference 600 plus electrochemical workstation (Gamry Instruments, Warminster, PA, USA). The working electrodes are the GCE and the auxiliary electrode is a Pt wire. The reference electrode is a saturated calomel electrode (SCE) that is connected to a salt bridge filled with 1 M LiAc.

For the potential response test, Ca^2+^-SC-ISEs were conditioned in 10^−4^ M CaCl_2_ overnight, then conditioned in 10^−7^ M CaCl_2_ for 3 h. The electromotive force (EMF) between the indicator and reference electrode was recorded in the target-ion solutions with concentrations ranging from 10^−7^ M to 10^−1^ M. Before the water-layer tests, the SC-ISEs were conditioned overnight in 0.1 M CaCl_2_. The water-layer tests were performed in 0.1 M CaCl_2_ for 2 h, followed by 0.1 M MgCl_2_ for 3 h, and finally 0.1 M CaCl_2_ for 12 h. The ion selectivity coefficients were measured via the separate solution method. In total, five mineral water and five soil-leaching solutions were tested by SC-ISEs and IC. The above potentiometric tests were performed using EMF 6 (Lawson Lab, Inc., Irvine, CA, USA). 

## 3. Results

### 3.1. Structure Comparison for NMC, RGO and CNT

**Figure 1:**
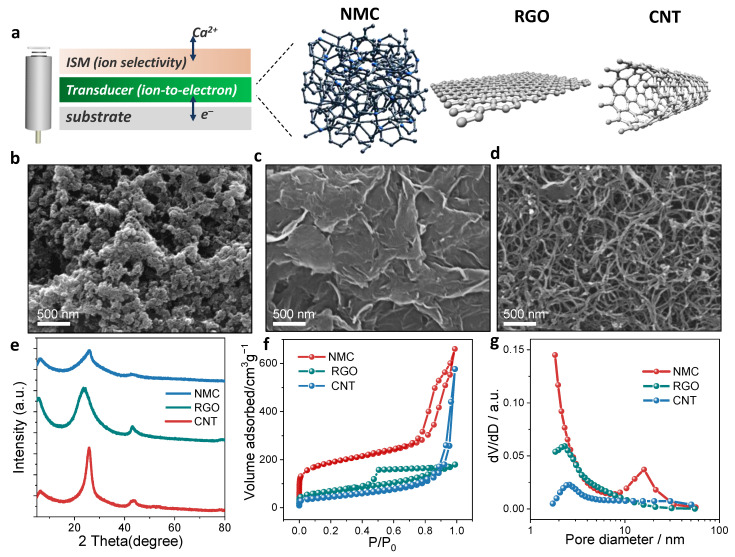
Carbon-based solid-contact ion-selective electrodes (SC-ISEs). (**a**) A schematic illustration of SC-ISEs based on SC materials of NMC, RGO and CNT. (**b**–**d**) SEM images of NMC, RGO and CNT. (**e**) XRD patterns for NMC, RGO and CNT. (**f**) N_2_ adsorption/desorption isotherms and (**g**) Pore-size distributions of NMC, RGO and CNT.

Figure 1a exhibits the basic structures of the SC-ISEs presenting a typical sandwich model including the solid contact for ion-to-electron signal transduction and ISM for ion recognition. As mentioned, the potential stability of SC-ISEs depends on the properties of the SC layer. In this work, three representative carbon-based SC materials, i.e., nitrogen-doped mesoporous carbon materials (NMC), reduced graphene oxide materials (RGO), and carbon nanotube materials (CNT) were proposed as the transduction layers of SC-ISEs (Figure 1b). The morphologies of the three carbon-based SC materials were examined via scanning electron microscopy (SEM) (Figure 1b–d). The NMC reveals an irregular and disorder structure, which forms a local porous characteristic. It is expected that the RGO and CNT show thin 2D planar and nanotubular structures, respectively. Further XRD patterns disclose the crystal structures of the three materials. The CNT exhibits sharp diffraction patterns, suggesting a well-defined crystal structure. The RGO shows a relatively broad diffraction peak at 2θ = 23.8°, which indicates the presence of carbon defects (Figure 1e). The NMC discloses the weakest crystal characteristic with a broad diffraction peak pattern and low intensity. This characteristic confirms the disordered structure of the NMC.

The specific surface areas of NMC, RGO and CNT were determined by the N_2_ adsorption–desorption isotherm (Figure 1f). NMC exhibits type I (H1) hysteresis loops of porous materials. The narrow and relatively uniform pore distribution discloses a typical mesoporous structure [56]. The H2 hysteresis loops appear in the RGO materials, which indicates more complex pore structures usually referred to as ink bottle pores. The volume of adsorbed gas increased at a relatively lower pressure, which demonstrates the existence of micropores. It is observed that CNT belongs to a typical physical adsorption disclosing the near-II adsorption isotherm with H3-type hysteresis loops [57]. The number of adsorbed molecules increase less with increasing gas pressure, which proves that the CNT is a porous solid with fine pores [58]. The Brunauer–Emmett–Teller (BET) results further show that the NMC has a specific surface area of 675.6 m^2^ g^−1^, much higher than RGO (240.9 m^2^ g^−1^) and CNT (158.5 m^2^ g^−1^). Additionally, it can also be seen that the NMC bears an advantage from the comparison of porosity test results (NMC of 0.84 cm^3^ g^−1^ > RGO of 0.26 cm^3^ g^−1^ ≈ CNT of 0.28 cm^3^ g^−1^). The pore diameter distribution is shown in Figure 1g. RGO and CNT are mainly composed of micropores with pore sizes of around 5 nm. However, the NMC is dominated by mesopores with sizes ranging from 10–30 nm. The mesoporous structure is favorable for ion transport, resulting in an improvement in efficiency of ion-to-electron transduction. 

**Figure 2:**
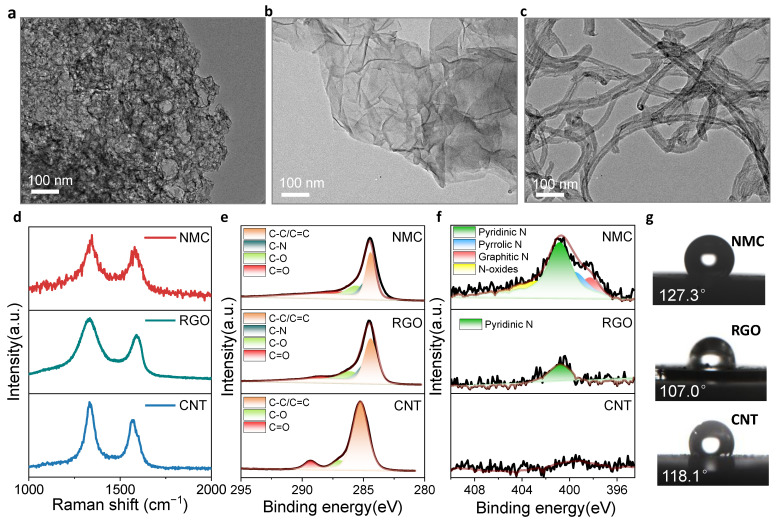
Structure comparison between NMC, RGO, CNT. (**a**–**c**) TEM images of NMC, RGO and CNT. (**d**) Raman spectra for NMC, RGO and CNT. (e-f) XPS spectra in the regions of (**e**) C 1s and (**f**) N 1s spectra for NMC, RGO and CNT. (**g**) Contact angles for NMC, RGO and CNT.

The microstructures are further examined via transmission electron microscopy (TEM). An abundance of mesopores are observed for the NMC (Figure 2a), which is consistent with the BET results (Figure 1g). RGO and CNT exhibit well-defined planar and tubular morphologies. The Raman spectra further examine their defects (Figure 2d). Typical D and G bands are observed between 1000–2000 cm^–1^. It is found that the ratios of the Raman intensity between the D band and G bands (*I*_D_/*I*_G_) follow the order, NMC (*I*_D_/*I*_G_ = 1.19) < CNT (*I*_D_/*I*_G_ = 1.34) < RGO (*I*_D_/*I*_G_ = 1.35), which proves that the NMC has relatively less defects compared with RGO and CNT. It should be noted herein that pores are not synonymous with defects. The perfect structure is the graphitic sp^2^ carbon while the exhibited defect represents a breakdown of the carbon structure at the atomic level, for example, heteroatomic doping. This will be illustrated by the following XPS results. The carbon defect demonstrated could affect the hydrophobicity. Less defects generally offer higher hydrophobicity. As shown in Figure 2g, the water-contact angles follow the order: NMC (127.3°) > CNT (118.1°) > RGO (107.0°). This result corresponds well with the Raman tests and confirm a relatively complete structure for the NMC. The hydrophobicity is a crucial parameter for the water-layer effect, which will be discussed in the next section.

The capacitance is affected by both porous structure and element doping for the carbon materials. In addition, the above discussed carbon defects have strong effects on the hydrophobicity. Therefore, XPS was used to further analyze the chemical bond information. The XPS spectra of the C 1s show two main doublets corresponding to sp^2^ and sp^3^ carbon bonds, respectively (Figure 2e). The typical oxygen-containing groups were observed in all three materials. However, the RGO showed the highest oxygen content, followed by CNT, and NMC. The less hydrophilic the oxygen-containing functional groups, the stronger the hydrophobicity [15]. This result is consistent with the contact angles. In addition, C-N groups appeared in both NMC and RGO. The N element in RGO originated from the precursor of GO since the HNO_3_ was used for the oxidation of graphite. Further N 1s spectra are shown in Figure 2f. The N 1s spectrum of NMC can be deconvoluted into four configurations, which are derived for pyridinic N, pyrrolic N, graphitic N, and N-oxides, respectively. However, the RGO shows a dominated pyridinic N configuration while no N element is observed in the CNT. Since nitrogen contains one more electron than carbon, local electron-rich sites are expected to appear as nitrogen atoms in the carbon matrix [59]. For example, pyridinic N and pyrrolic N contribute one and two p-electrons to the carbon matrix, respectively, thus pyridinic N and pyrrolic N increase the specific capacitance [59], which will be confirmed in conjunction with the capacitance test in the following section. Overall, the above results prove that NMC has the highest specific surface area with mesoporous structure and abundant N-doping, which would be beneficial to improving the EDL capacitance and ion-to-electron transduction. 

### 3.2. Capacitance Evaluation

**Figure 3:**
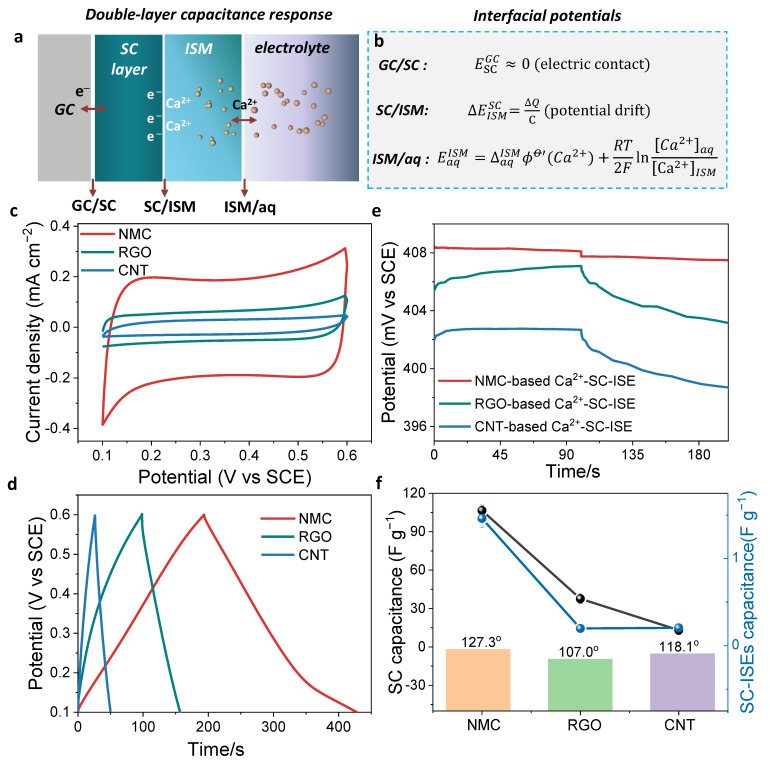
Capacitance evaluation. (**a**) Electric-double-layer (EDL) capacitance-based SC-ISEs which contain three phase boundaries including GC/SC, SC/ISM, and ISM/aq interfaces and (**b**) the corresponding phase interfacial potentials. (**c**) Cyclic voltammetry curves for SC materials (NMC, RGO, CNT) in 0.1 M CaCl_2_. (**d**) Corresponding charge–discharge tests of SC materials under a current density of 0.25 A g^−1^. (**e**) Chronopotentiometry tests for the corresponding Ca^2+^-SC-ISEs based on NMC, RGO and CNT SC materials. (**f**) Summary of the capacitances of the SC materials and SC-ISEs and correlation with hydrophobicity (contact angles). The error bars for the SC-ISEs chronopotentiometry tests were calculated using three individual electrodes.

Following the discussion of the structure and composition for the three carbon-based SC materials, their capacitances will be examined in this section. Figure 3a exhibits the ion response mechanisms for the EDL capacitance-type SC-ISEs. Three phase boundaries of GC/SC, SC/ISM, and ISM/aqueous solution (ISM/aq) interfaces are composed. The EMF equates the sum of potentials at these three interfaces. The potential at the GC/SC interface is close to zero since most EDL capacitance-based SC materials have high electrical conductivity (electric contact) (Figure 3b). The potential at the ISM/aq interface is determined by the Nernstian equation, which depends on the activity of target ions. Therefore, the potential stability for EDL-capacitance-type SC-ISEs strongly relies on the potential variation at the SC/ISM interface. However, this interfacial potential could not be defined because there is no charge transfer reaction. It can only determine the potential variation, which is represented by $\Delta E_{ISM}^{SC}$=∆Q/C, where ∆Q is the passed charge and C is the EDL capacitance. Therefore, capacitance is a decisive factor in maintaining the potential stability of SC-ISEs. For this purpose, the capacitance of three SC materials were examined in detail.

Figure 3 shows the CVs of three types of SC materials in aqueous (0.1 M CaCl_2_). It can be clearly observed that the NMC has the largest current density, much higher than RGO and CNT, indicating a higher capacitance. It should be noted that the mass loading is the same for the three carbon-based SC materials (1 mg cm^–2^). Galvanostatic charge–discharge tests were used to further evaluate their specific capacitance at a current density of 0.25 A g^–1^ (Figure 3d). Obviously, the charge–discharge time of NMC is longer than that of RGO and CNT, which demonstrates that NMC can store more charge. The specific capacitance (C_s_) is calculated by the equation: C_s_ = *I* × Δt/ΔV, where *I* is the applied current density (0.25 A g^–1^); Δt is the average time for the charge and discharge, and ΔV is the potential window. The calculated C_s_ follows the order: NMC (106.6 F g^−1^) > RGO (37.7 F g^−1^) > CNT (13.1 F g^−1^). It is found that the capacitance of NMC is much higher than that of RGO and CNT.

After evaluation of the capacitance of SC materials, the capacitance of fabricated Ca^2+^-SC-ISEs were further evaluated through chronopotentiometry under the application of a current at ±1 mA (Figure 3e). The SC-ISEs were prepared by coating the SC-modified GCE with Ca^2+^-ISM (see experimental section for details). The specific capacitance was determined: NMC (1.461 F g^−1^) > CNT (0.204 F g^−1^) ≈ RGO (0.197 F g^−1^). The NMC-based Ca^2+^-SC-ISE again demonstrates the highest capacitance. The results for specific capacitance and hydrophobicity are summarized in Figure 3f. The NMC shows the highest capacitance and strongest hydrophobicity. Based on the above analysis, the mesoporous structure and N-doping are beneficial to increasing the capacitance thus allowing inhibition of the polarization effect of the external current on the electrode.

### 3.3. Analytical Performances

**Figure 4:**
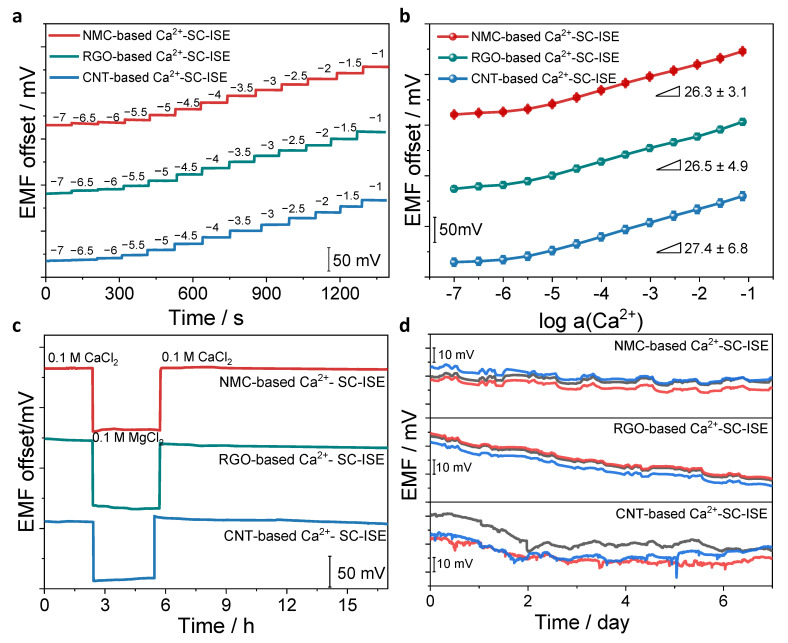
Performance comparison of NMC-, RGO- and CNT-based Ca^2+^-SC-ISEs. (**a**) Potential response measurements through increasing the concentration of Ca^2+^ in the solution. (**b**) Linear calibration curves of Ca^2+^ responses. (**c**) Water-layer tests. (**d**) Long-term stability monitoring for 7 days. The potential drifts were calculated according to the potential differences between the maximal and minimal potentials during the whole test time. The error bars reflect the results of three individual measurements.

After evaluating capacitance, the potentiometric responses of the Ca^2+^-SC-ISEs based on three SC materials were further examined (Figure 4a,b). The sensitivity and limit of detection (LOD) for NMC-based, RGO-based and CNT-based Ca^2+^-SC-ISEs were determined as 26.3 ± 3.1 mV dec^−1^/10^−5.5^ M, 26.5 ± 4.9 mV dec^−1^/10^−5.6^ M, and 27.4 ± 6.8 mV dec^−1^/10^−5.6^ M, respectively. Three types of Ca^2+^-SC-ISEs exhibit nearly Nernstian responses. There are little differences in the sensitivity and LOD because the primary response is mainly dependent on the ISM. 

The formation of water layer at the SC/ISM interface cause potential drifts and even leads to the failure of ISM. Water-layer tests were conducted through the separation solution method (Figure 4c). It is found that the RGO-based Ca^2+^-SC-ISE and CNT-based Ca^2+^-SC-ISE show obvious potential drifts in the second stage (solution was changed from 0.1 M CaCl_2_ to 0.1 M MgCl_2_) and the third stage (back to 0.1 M CaCl_2_). However, the potential of NMC-based Ca^2+^-SC-ISE keeps stable in the three stages, which indicates no water layer existed. According to the above discussion, CNT and RGO exhibit insufficient capacitance and hydrophobicity. Therefore, both exhibit similar water-layer results. NMC has good capacitance and hydrophobicity, thus avoiding the water-layer effect. The above results suggest that the effects of capacitance and hydrophobicity should be reasonably considered in the development of SC materials. Figure 4d demonstrates a long-term potential stability. The potential drifts over 7 days were 66.9 ± 14.5 μV h^−1^ (NMC), 189.8 ± 3.4 μV h^−1^ (RGO) and 160.3 ± 31.9 μV h^−1^ (CNT), respectively. These results confirm the superiority of NMC as an ion-to-electron transducer layer, which stems from the high capacitance and hydrophobicity. Compared with other studies for the detection of Ca^2+^, the NMC-based SC-ISEs have comparable sensitivity, detection limit and stability (Table S1). Significantly, the NMC has relatively excellent stability in carbon-based SC-ISEs, which may provide guidance for the preparation of carbon-based SC-ISEs.

### 3.4. Calcium Detection in Real Samples

The calcium ion is an essential element that affects the natural circulation and metabolism in the human body [60]. Determination of the calcium ion plays an important role in the clinical diagnosis of various diseases such as fractures and hypercalcemia. Moreover, calcium ions also play an important structural role in the cell wall and membrane as counter-cations of inorganic and organic anions. Additionally, calcium deficiency or calcium excess will lead to a costly dysregulation in horticulture. Therefore, calcium detection has great social value and scientific significance [61,62].

**Figure 5:**
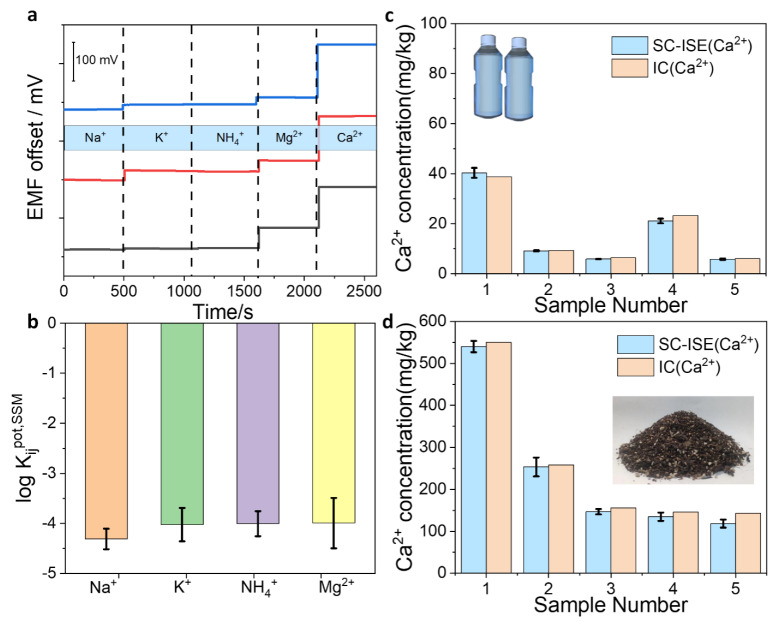
Calcium detection in real samples. (**a**) Potential measurements at a concentration of 0.1 M for other interfering ions (Na^+^, K^+^, NH_4_^+^, Mg^2+^) and target ion (Ca^2+^) by three individual measurements which are presented in different colors. (**b**) Selectivity coefficients obtained via the separation solution method. (**c**) Calcium-ion detection in mineral water and (**d**) soil-leaching solutions via the SC-ISEs and Ion Chromatography (IC).

In this work, a proof-of-concept application of Ca^2+^ ion analysis in mineral water and soil-leaching solutions was demonstrated by using NMC-based SC-ISEs. The above results demonstrate the NMC-based Ca^2+^-SC-ISE shows better analytical performances, and the selectivity was first examined by using the separation solution method. The potentials in target Ca^2+^ ions and interfering ions were separately measured (Figure 5a). Apparently, the sensor shows the highest potential for Ca^2+^, indicating good selectivity. Corresponding selectivity coefficients (log *K*_ij_) were further calculated via the equation $logK_{i,j}^{pot}=\frac{\left(E_{j}-E_{i}\right)Z_{i}F}{2.303RT}+log\frac{a_{i}}{a_{j}^{z_{i}/z_{j}}}$ (where *E*_i_ and *E*_j_ are the potentials in the target and interfering ions, respectively). The results are presented in Figure 5b. It is found that the log *K*_ij_ values toward interfering ions are all less than –3, which confirms that the sensor can be used for the detection of actual samples. Moreover, the Ca^2+^ concentration in five kinds of mineral waters and soil-leaching solutions was tested with the prepared sensors. Each sample was assessed by three individual Ca^2+^-SC-ISEs. The detected results were compared with IC, and the results are shown in Figure 5c,d. As can be seen, the two measurement results are basically consistent, proving that the ion-selective electrode can be used for practical sample analysis. In short, the fabricated sensors can quickly and accurately detect Ca^2+^ levels in drinking water and soil solutions. In the future, these promising SC-ISEs can be combined with electronic integration technology and internet-based software to build an ion-monitoring platform.

## 4. Conclusions

Three representative carbon-based SC materials including NMC, RGO, and CNT and the effect on the performances of Ca^2+^-SC-ISEs were examined in detail. It was demonstrated that the interfacial capacitance can be improved effectively via mesoporous and N-doping engineering. The mesopore structure allows rapid diffusion of ions and an increased specific surface area. Therefore, NMC with both high capacitance and hydrophobicity results in the effective inhibition of water-layer formation. The potentiometric Ca^2+^ sensors fabricated based on NMC show a Nernstian slope, excellent reproducibility and good potential stability. The prepared SC-ISEs were used for the ion analysis of mineral water and soil-leaching solution. This work demonstrates mesoporous and element-doping engineering as an effective strategy to enhance the interfacial capacitance and further improve the potential stability of SC-ISEs.

## Data Availability

The data are available upon reasonable request from the corresponding author.

## Supplementary Materials

The following supporting information can be downloaded at: https://www.mdpi.com/article/10.3390/membranes12090903/s1, Table S1: Comparison of the potentiometric performance of the NMC-based SC-ISEs with the reported Ca^2+^-SC-ISEs. References [26,61,62,63,64,65] have been cited in Supplementary Materials.
