# Supplementary material for: Carbon-Based Transducers for Solid-Contact Calcium Ion-Selective Electrodes: Mesopore and Nitrogen-Doping Effects
Source: Membranes (Basel). 2022 Sep 19;12(9):903. doi: 10.3390/membranes12090903 (PMC9505166; doi:10.3390/membranes12090903)
Supplement: Supplementary file 1 [file membranes-12-00903-s001.zip › membranes-1889809-supplementary.pdf]

membranes

MDPI

Supplementary Materials

Carbon-Based Transducers for Solid-Contact Calcium Ion-Selective Electrodes: Mesopore and Nitrogen-Doping Effects

Yirong Zhang, Yitian Tang, Rongfeng Liang, Lijie Zhong *, Jiexian Xu, Huici Lu, Xiaofeng Xu, Tingting Han, Yu Bao, Yingming Ma, Shiyu Gan and Li Niu *

Table S1. Comparison of the potentiometric performances of the NMC-based SC-ISEs with the reported Ca^{2+}-SC-ISEs.

Solid-Contact Materials	Slope	LOD	Target Ion	Stability	Short-Term Stability	Capacitance	Reference
single-walled carbon nanotubes (SWCNTs)	28.7 mV dec^{-1}	0.631 μM	Ca^{2+}	493 μV h^{-1}.	930 μV s^{-1}	5.37 μF	1
single-walled carbon nanohorns (SWCNHs)	29.69 mV dec^{-1}	0.794 μM	Ca^{2+}	/	43 μV s^{-1}	23.26 μF	2
Ag@AgCl/1-tetradecylmethylimidazolium chloride	28.3 mV dec^{-1}	/	Ca^{2+}	/	13.3 μV s^{-1}	75.2 μF	3
MXene ($Ti_3C_2T_x$)	26.4 mV dec^{-1}	0.794 μM	Ca^{2+}	/	4.0 μV s^{-1}	250 μF	4
MXene (Ti_2CT_x)	24.9 mV dec^{-1}	1.0 μM	Ca^{2+}	/	5.0 μV s^{-1}	200 μF	4
$NiCo_2S_4$ microsphere ($NiCo_2S_4$-EtOH)	27.5 mV dec^{-1}	0.5 μM	Ca^{2+}	6.4 ± 0.3 μV h^{-1}	1.77 μV s^{-1}	565 μF	5
Black phosphorous	28.3 mV dec^{-1}	0.4 μM	Ca^{2+}	/	72 μV s^{-1}	/	6
nitrogen-doped mesoporous carbon (NMC)	26.3 mV dec^{-1}	3.16 μM	Ca^{2+}	66.9 ± 14.5 μV h^{-1}	17.17 μV s^{-1}	58.2 μF	This work
reduced graphene oxide (RGO)	26.5 mV dec^{-1}	2..51 μM	Ca^{2+}	189.8 ± 3.4 μV h^{-1}	127.17 μV s^{-1}	7.9 μF	This work
carbon nanotubes (CNT)	27.4 mV dec^{-1}	2..51 μM	Ca^{2+}	160.3 ± 31.9 μV h^{-1}	123.08 μV s^{-1}	8.1 μF	This work

References

1. Hernandez, R.; Riu, J.; Rius, F. X., Determination of calcium ion in sap using carbon nanotube-based ion-selective electrodes. *Analyst* **2010**, *135*, 1979-1985.
2. Jiang, C. M.; Yao, Y.; Cai, Y. L.; Ping, J. F., All-solid-state potentiometric sensor using single-walled carbon nanohorns as transducer. *Sens. Actuator B-Chem*. **2019**, *283*, 284-289.
3. Zeng, X. Z.; Qin, W., A solid-contact Ca^{2+}-selective electrode based on an inorganic redox buffer of Ag@AgCl/1-tetradecyl-3-methylimidazolium chloride as ion-to-electron transducer. *Talanta* **2020**, *209*, 120570.
4. Shao, Y. Z.; Yao, Y.; Jiang, C. M.; Zhao, F. N.; Liu, X. X.; Ying, Y. B.; Ping, J. F., Two-dimensional MXene nanosheets (types Ti3C2Tx and Ti2CTx) as new ion-to-electron transducers in solid-contact calcium ion-selective electrodes. *Microchim. Acta* **2019**, *186*, 750.
5. Li, Y. H.; Li, J. H.; Qin, W., All-Solid-State Polymeric Membrane Ion-Selective Electrodes Based on NiCo(2)S(4)as a Solid Contact. *Anal. Chem.* **2022**, *94*, 3574-3580.
6. Kou, L. J.; Fu, M. L.; Liang, R. N., Solid-contact Ca^{2+}-selective electrodes based on two-dimensional black phosphorus as ion-to-electron transducers. *RSC Adv*. **2017**, *7*, 43905-43908.
